# Caffeic Acid Phenethyl Ester Regulates PPAR's Levels in Stem Cells-Derived Adipocytes

**DOI:** 10.1155/2016/7359521

**Published:** 2016-01-24

**Authors:** Luca Vanella, Daniele Tibullo, Justyna Godos, Francesca Romana Pluchinotta, Claudia Di Giacomo, Valeria Sorrenti, Rosaria Acquaviva, Alessandra Russo, Giovanni Li Volti, Ignazio Barbagallo

**Affiliations:** ^1^Department of Drug Science, Biochemistry Section, University of Catania, Viale Andrea Doria 6, 95125 Catania, Italy; ^2^Department of Surgery and Medical Specialties, Section of Hematology, University of Catania, Via Salvatore Citelli 6, 95124 Catania, Italy; ^3^IRCCS “S. Donato” Hospital, San Donato Milanese, Piazza Edmondo Malan, 20097 Milan, Italy; ^4^Department of Biomedicine and Biotechnologies, University of Catania, Viale Andrea Doria 6, 95125 Catania, Italy

## Abstract

Hypertrophic obesity inhibits activation of peroxisome proliferators-activated receptor gamma (PPAR*γ*), considered the key mediator of the fully differentiated and insulin sensitive adipocyte phenotype. We examined the effects of Caffeic Acid Phenethyl Ester (Cape), isolated from propolis, a honeybee hive product, on Adipose Stem Cells (ASCs) differentiation to the adipocyte lineage. Finally we tested the effects of Cape on insulin-resistant adipocytes. Quantification of Oil Red O-stained cells showed that lipid droplets decreased following Cape treatment as well as radical oxygen species formation. Additionally, exposure of ASC to high glucose levels decreased adiponectin and increased proinflammatory cytokines mRNA levels, which were reversed by Cape-mediated increase of insulin sensitivity. Cape treatment resulted in decreased triglycerides synthesis and increased beta-oxidation. Exposure of ASCs to Lipopolysaccharide (LPS) induced a reduction of PPAR*γ*, an increase of IL-6 levels associated with a well-known stimulation of lipolysis; Cape partially attenuated the LPS-mediated effects. These observations reveal the main role of PPAR*γ* in the adipocyte function and during ASC differentiation. As there is now substantial interest in functional food and nutraceutical products, the observed therapeutic value of Cape in insulin-resistance related diseases should be taken into consideration.

## 1. Introduction

White adipose tissue is constituted by different cellular types including preadipocytes, mature adipocytes, fibroblasts, pericytes, macrophages, neutrophils, lymphocytes, endothelial cells, and Adipose Stem Cells (ASCs). The balance between these different cell types and their expression profile is closely related to maintenance of the organ metabolic function [1]. Additionally, adipose tissue expresses numerous receptors that allow it to respond to afferent signals from traditional hormone systems as well as the central nervous system; thus, adipose organ mediates numerous physiological and pathological processes by factors that control glucose metabolism, appetite, immunological responses, inflammatory responses, angiogenesis, blood pressure regulation, and reproductive function.

Adipocytes produce a variety of molecules biologically active including interleukins, tumor necrosis factor-alpha (TNF-*α*), resistin, leptin, adiponectin, monocyte chemoattractant protein- (MCP-) 1, transforming growth factor- (TGF-) *β*, insulin-like growth factor- (IGF-) 1, and C-reactive protein (CRP); a deregulated production of these factors is involved in the systemic inflammatory process occurring in obesity [2, 3].

Obesity is a chronic condition defined by an excess amount body fat; it is no longer considered to be a cosmetic problem but a risk factor for several metabolic disorders [4–6] in particular to visceral adipose tissue is associated with numerous clinical disorders such as hypertension, hyperglycemia, insulin resistance, endothelial dysfunction, elevated triglycerides, and high cholesterol levels [7–10].

The uncontrolled hypertrophy of adipose tissue is characterized by the infiltration of macrophages, prominent source of proinflammatory cytokines, such as TNF-*α* and IL-6, that can block insulin signaling in adipocytes, providing a potential link between inflammation and insulin resistance [11, 12]. Adipose tissue growth involves formation of new adipocytes from precursor cells, further leading to an increase in adipocyte size. Moreover, adipose tissue expansion is associated with adipocyte dysfunction and increased inflammatory processes.

The transition from undifferentiated fibroblast-like preadipocytes into mature adipocytes constitutes the adipocyte life cycle, and treatments that regulate both size and number of adipocytes may provide a better therapeutic approach for treating obesity.

Current studies on obesity focus on discovering plant active components that have the capability to suppress the differentiation of mesenchymal stem cells (MSCs) and preadipocytes into hypertrophic adipocytes. Recent studies reported that Caffeic Acid Phenethyl Ester (Cape), isolated from propolis, a honeybee hive product, suppresses 3T3-L1 murine cells differentiation into adipocytes [13, 14].

Additionally, it has been demonstrated that Cape completely blocks production of ROS and xanthine oxidase system (XO) and also reduces malondialdehyde level secondary to polyunsaturated fatty acid oxidation [15]. Cape inhibits cyclooxygenase, suppresses activity of lipoxygenase, and prevents lipid peroxidation [16, 17]. Cape is a potent and specific inhibitor of activation of nuclear transcription factor NF-*κ*B by TNF-*α* [18].

Despite some data demonstrating that Cape inhibits murine preadipocyte differentiation, its anti-inflammatory effects on human ASCs adipocyte differentiation and hypertrophic adipocytes are not known. The aim of this study is to investigate the molecular mechanism of Cape, a strong antioxidant, to prevent adipogenesis but also to restore the function of inflamed mature adipocytes.

## 2. Material and Methods

### 2.1. Adipose Stem Cells Isolation and Culture

Subcutaneous adipose tissue sample was obtained from a healthy patient that underwent abdominal plastic surgery (male, 23 years old, 98 kg b.w.); the subject provided his written consent before inclusion in the study. Since this is a nontherapeutic trial, it was carried out with the consent of the subject legally acceptable according our Italian Government (Legge 675/1996 and DL 196/2003, art. 40. Art 32 Codice Italiano di Deontologia Medica).

Adipose tissue was minced with scissors and scalpels into less than 3 mm pieces and isolation of ASCs proceeded as previously described [19]. Briefly, after gentle shaking with equal volume of PBS, the mixture separated into two phases. The upper phase (containing stem cells, adipocytes, and blood) after washing with PBS was enzymatically dissociated with 0.075% collagenase (type I)/PBS for 1 h at 37°C with gentle shaking. The dissociated tissue was then mixed with an equal volume of DMEM (GIBCO-BRL, Japan) supplemented with 10% FBS and incubated 10 min at room temperature. The solution then was separated into two phases. The lower phase was centrifuged at 1500 rpm for 5 min at 20°C. The cellular pellet was resuspended in 160 mM NH_4_Cl to eliminate erythrocytes and passed through a 40 *μ*m mesh filter into a new tube. The cells were resuspended in an equal volume of DMEM/10% FBS and then centrifuged. Isolation resulted in obtaining 7.7 × 10^6^ of adherent cells for a primary culture from 5 g of adipose tissue (approximately 1.0 × 10^5^ to 4.6 × 10^6^/1 g) after 7 to 10 days of culture. The cells were suspended in DMEM/10% FBS plated in concentration of 1–5 × 10^6^ cells/75 cm^2^.

### 2.2. Detection of ASCs Cell Markers by FACS Analysis

The phenotype of ASCs was evaluated by flow-cytometry analysis (FC500 Beckman Coulter). The ASCs presented as a homogeneous fibroblastic cell population. Flow cytometric analysis of passage 4th cells revealed that cells were negative for CD34 and CD45 and that cells were positive for CD105 and CD90.

### 2.3. Differentiation of Human ASCs into Adipocytes

ASCs (passages 4 to 5) were plated in a 75 cm^2^ flask at a density of 1 to 2 × 10^4^ cells and cultured in DMEM with 10% FBS for 7 days. The medium was replaced with adipogenic medium, and the cells were cultured for additional 14 or 21 days.

The adipogenic media (Lonza, Basel, SW) consisted of complete culture medium supplemented with DMEM-high glucose, 10% (v/v) FBS, 10 *μ*g/mL insulin, 0.5 mM dexamethasone (Sigma-Aldrich, St. Louis, MO), 0.5 mM isobutylmethylxanthine (Sigma-Aldrich, St. Louis, MO), and 0.1 mM indomethacin (Sigma-Aldrich, St. Louis, MO). Media were changed every 3 days. Our preliminary results on ASCs demonstrate no significant differences in cell viability between 5, 10, and 25 *μ*M of Cape. In our experiments human ASCs were cultured in the presence of Cape (10 *μ*M) which was administered every 3 days in the first set of experiments while for the second set of experiments Cape was added once for the last 3 days of differentiation at the same concentration. Additionally, ASC-derived adipocytes were cultured in adipogenic differentiation media and LPS was added 6 hrs before collecting cells at a dose of 1 ng/mL.

### 2.4. ROS Measurement

Determination of ROS was performed by using a fluorescent probe 2′,7′-dichlorofluorescein diacetate (DCFH-DA). The fluorescence (corresponding to the oxidized radical species 2′,7′-dichlorofluorescein (DCF)) was monitored spectrofluorometrically (excitation, *λ* = 488 nm; emission, *λ* = 525 nm). The total protein content was evaluated for each sample, and the results are reported as increase in fluorescence intensity (IFM).

### 2.5. Oil Red O Staining

Staining was performed using 0.21% Oil Red O in 100% isopropanol (Sigma-Aldrich, St. Louis, MO, USA). Briefly, adipocytes were fixed in 10% formaldehyde, stained with Oil Red O for 10 minutes, and rinsed with 60% isopropanol (Sigma-Aldrich), and the Oil Red O was eluted by adding 100% isopropanol for 10 minutes, and the optical density (OD) was measured at 490 nm, for 0.5 sec reading. Nuclei were stained with NucBlue (Life Technologies, NY). Lipid droplets accumulation was examined by using inverted multichannel LED fluorescence microscope (Evos, Life Technologies, NY).

### 2.6. RNA Extraction and qRT-PCR

RNA was extracted by Trizol reagent (Invitrogen, Carlsbad, CA, USA). First strand cDNA was then synthesized with Applied Biosystem (Foster City, CA, USA) reverse transcription reagent.

Quantitative real-time PCR was performed in 7900HT Fast Real-Time PCR System Applied Biosystems using the SYBR Green PCR MasterMix (Life Technologies). The primer sequences used are shown in Table 1. The specific PCR products were detected by the fluorescence of SYBR Green, the double stranded DNA binding dye. The relative mRNA expression level was calculated by the threshold cycle (Ct) value of each PCR product and normalized with that of GAPDH by using comparative 2^−ΔΔCt^ method.

### 2.7. Statistical Analyses

Statistical significance (*p* < 0.05) of differences between experimental groups was determined by the Fisher method for analysis of multiple comparisons. For comparison between treatment groups, the null hypothesis was tested by either single-factor analysis of variance (ANOVA) for multiple groups or the unpaired *t*-test for two groups, and the data are presented as mean ± standard error (SE).

## 3. Results

### 3.1. Determination of ASCs Surface Markers Expressions

**Figure 1:**
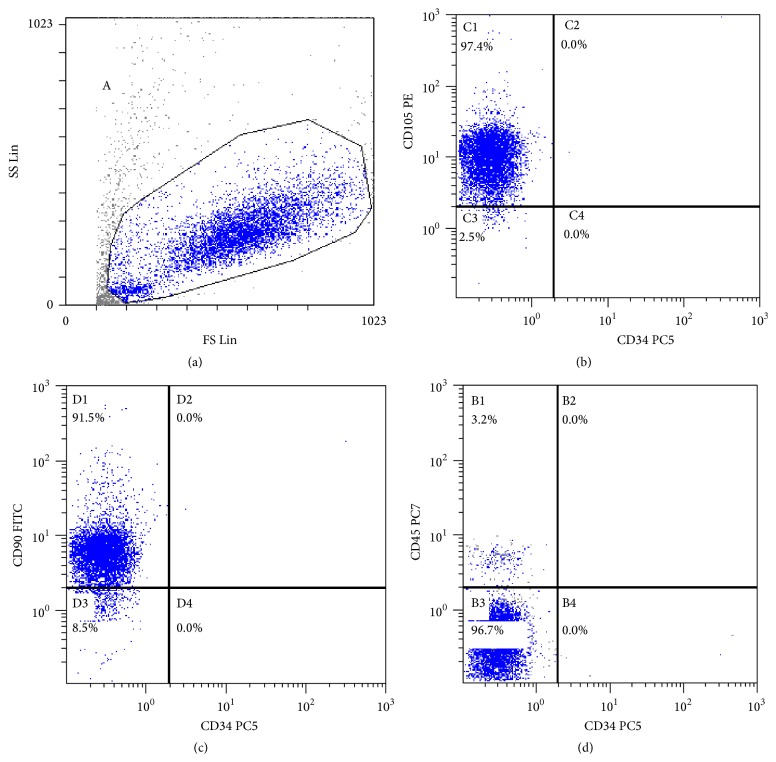
Determination of ASCs phenotype. Membrane antigen expressions of CD45, CD34, CD90, and CD105 on ASCs were analyzed by FACS analysis. As negative markers, CD45 (common lymphocytes antigen) and CD34, an hematopoietic stem cell marker, were shown to be low expressed.

ASCs were obtained from human adipose tissue and were cultured in adipocyte-induced medium. Confirmation of the ASC phenotype was made by the presence of positive markers measured by FACS: 97.4% of the cells were positive for CD105 (Figure 1(b)) and 91.5% of the cells were positive for CD90 (Figure 1(c)). In addition, the low percentages of CD34 (Figure 1(a)), a hematopoietic stem cell marker, and CD45, a lymphocytic marker (Figure 1(d)), indicate that the ASCs were not significantly contaminated with lymphocytes and hematopoietic stem cells.

### 3.2. Temporal Sequence of Adipogenic Marker Expression

To investigate signals that might regulate the differentiation of ASCs, we first conducted a time course study focusing on days 0, 7, 18, and 21 of adipogenesis.

**Figure 2:**
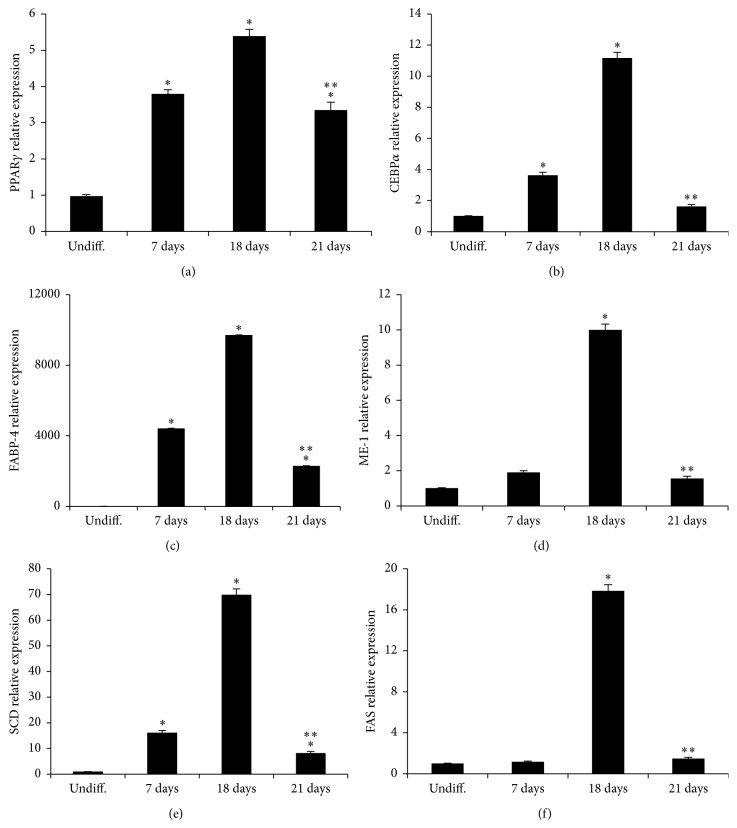
Expression of adipogenic markers during adipogenesis. qRT-PCR revealed a marked increase of PPAR*γ*, CEBP*α*, FABP4, ME1, SCD, and FAS at 18 days of adipogenic differentiation. Values represent the means ± SD of 4 experiments performed in triplicate. ^*∗*^ *p* < 0.05, significant result versus undifferentiated cells. ^*∗∗*^ *p* < 0.05 significant result versus 18 days.

**Figure 3:**
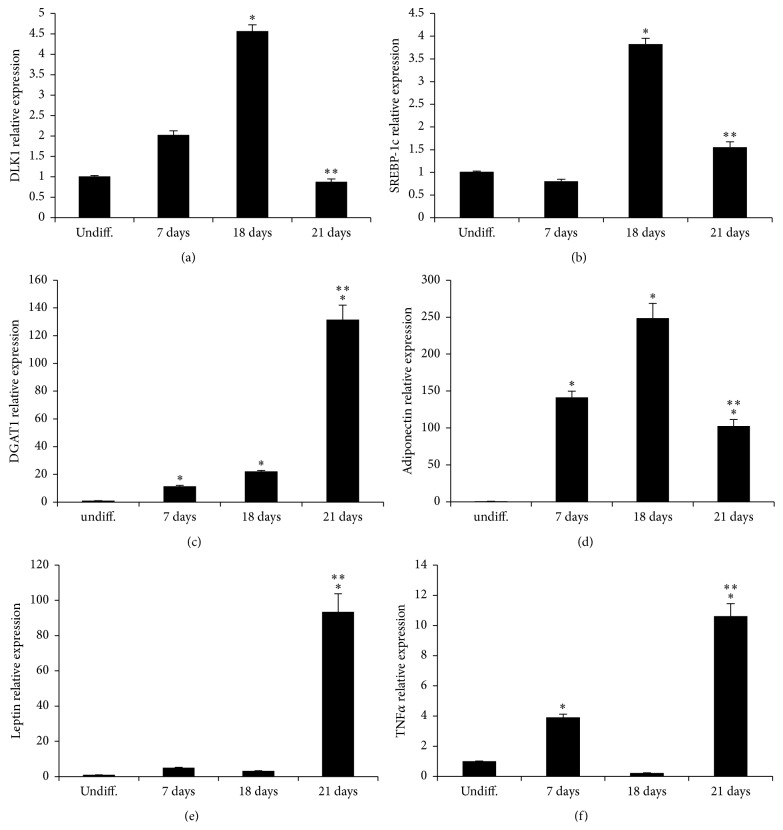
Expression of adipogenic markers and cytokines levels during adipogenesis. qRT-PCR revealed a marked increase of DLK1, SREBP-1c, DGAT1, adiponectin, leptin, and TNF*α*. Values represent the means ± SD of 4 experiments performed in triplicate. ^*∗*^ *p* < 0.05, significant result versus undifferentiated cells. ^*∗∗*^ *p* < 0.05 significant result versus 18 days.

The mRNA levels of PPAR*γ*, CEBP*α*, FABP4, DLK1, SREBP-1c, ME1, SCD, and FAS increased severalfold following differentiation. However, after 21 days of differentiation mRNA levels were significantly lower compared to 18-day differentiated adipocytes (Figures 2 and 3).

Similarly, adiponectin expression was reduced in 21-day differentiated adipocytes while leptin and TNF*α* were significantly increased after 21 days (Figure 3).

The increase of lipid droplets mirrored the increase in triglycerides accumulation consequence of increased levels of DGAT1 mRNA levels (Figure 3(c)).

### 3.3. The Effect of Cape on Adipogenesis and ROS Production

**Figure 4:**
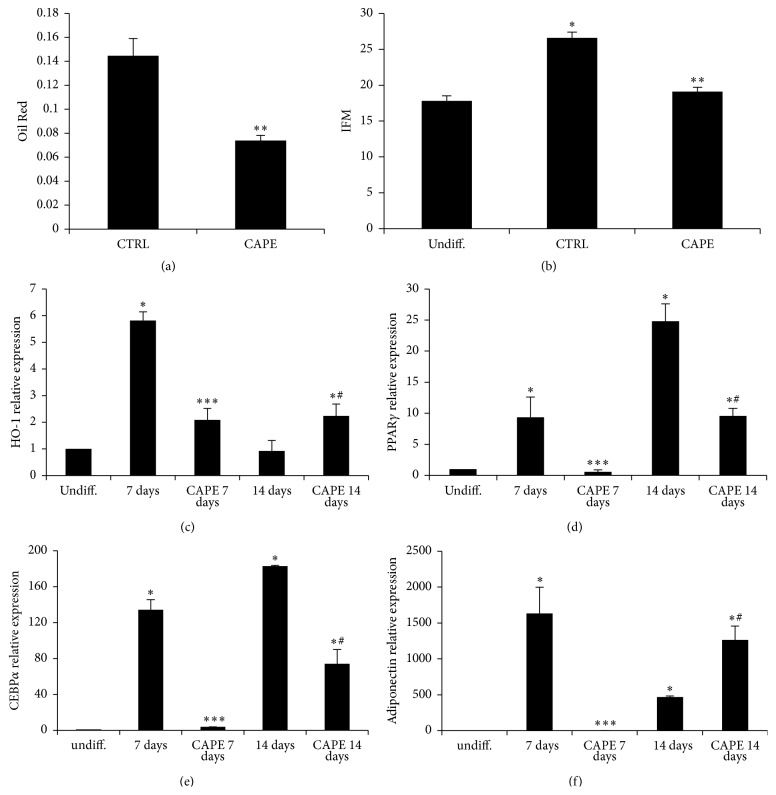
The effect of Cape on differentiation. (a) Lipid droplets area was determined by Oil red O staining after 14 days. (b) Intracellular oxidants in undifferentiated, differentiated untreated, and treated cells for 14 days with Cape (10 *μ*M). (c–f) Effect of Cape on HO-1 PPAR*γ*, CEBP*α*, and adiponectin mRNA levels after 7 and 14 days of differentiation. Values represent the means ± SD of 4 experiments performed in triplicate. ^*∗*^ *p* < 0.05, significant result versus undifferentiated cells. ^*∗∗*^ *p* < 0.05, significant result versus control cells. ^*∗∗∗*^ *p* < 0.05, significant result versus 7 days. ^#^ *p* < 0.05, significant result versus 14 days.

In another set of experiments, we examined the effect of Cape on lipid accumulation after 14 days, using standard culture conditions by measuring Oil Red O-stained lipid droplet area (Figure 4(a)). Quantification of Oil Red O-stained cells showed that lipid droplets decreased following Cape treatment. This result was supported by the strong reduction of ROS formation induced by Cape after 14 days of differentiation (Figure 4(b)). In addition to that, the expression of the antioxidant enzyme HO-1 in the presence of Cape significantly increased after 14 days (Figure 4(c)), which was consistent with our previous results showing decreased lipogenesis. To further examine the mechanism by which Cape regulates the adipogenic cell differentiation we measured PPAR*γ*, CEBP*α*, and adiponectin mRNA levels in adipocytes. As seen in Figures 4(d)–4(f), PPAR*γ* and CEBP*α* levels were significantly (*p* < 0.05) increased in mature adipocytes (14 days of ASC-derived adipocyte differentiation) and conversely, adiponectin levels were declining (*p* < 0.05) at day 14. The increase in PPAR*γ* and CEBP*α* mRNA in preadipocytes (7 days) and mature adipocytes was prevented by Cape treatment. In contrast, Cape significantly increased adiponectin in ASC-derived adipocytes (*p* < 0.05) (Figure 4(f)).

### 3.4. Effect of Cape on Functional Activity of Adipocytes

**Figure 5:**
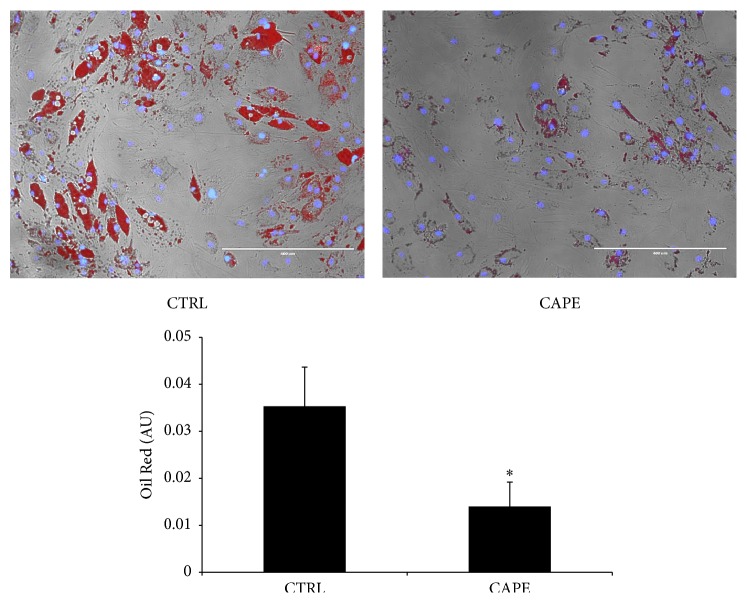
Pictures of lipid droplets of a representative sample in the absence or presence of Cape. Adipogenesis was measured as the relative absorbance of Oil Red O at day 21 after inducing adipogenesis as described in materials and methods (mean ± SD, ^*∗*^ *p* < 0.05 versus control). Values represent the means ± SD of 4 experiments performed in triplicate.

**Figure 6:**
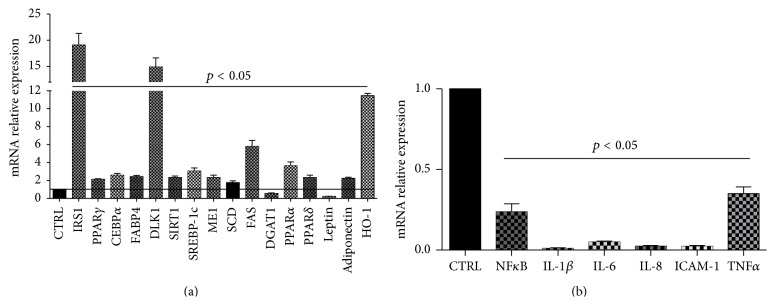
Effect of Cape on cytokines and adipogenic markers. Gene levels are expressed as fold of increase compared to the respective control. Cape was added once after 18 days of differentiation at the concentration of 10 *μ*M. Values represent the means ± SD of 4 experiments performed in triplicate. ^*∗*^ *p* < 0.05, significant result versus differentiated cells.

To investigate the effects of Cape on functionality of fully differentiated adipocytes, we measured several adipogenic markers. ASCs isolated from the subcutaneous adipose tissue of a human donor were exposed to Cape only at the end of differentiation for 3 days. Our results show that Oil Red O staining was decreased after Cape treatment compared to untreated ASCs-derived adipocytes (Figure 5). Cape treatment slightly reduced DGAT1 mRNA levels and upregulated the levels of HO-1, adiponectin, IRS1, PPAR*γ*, CEBP*α*, FABP4, DLK1, SIRT1, SREBP-1c, ME1, SCD, and FAS compared with cells treated with vehicle solution (Figure 6(a)). In addition, we examined markers of beta-oxidation such as PPAR*α* and PPAR*δ* mRNA and showed that Cape treatment increased both mRNA levels (Figure 6(a)). In contrast, leptin, NF*κ*B, IL-1*β*, IL-8, ICAM-1, and TNF*α* levels were reduced in Cape treated cells (Figure 6(b)).

### 3.5. Effect of Cape on LPS Stimulated Adipocytes

To analyze the effect of LPS on inflammation and glucose metabolism in fully differentiated adipocytes, we treated cells with LPS (1 ng/mL) for 6 hr.

**Figure 7:**
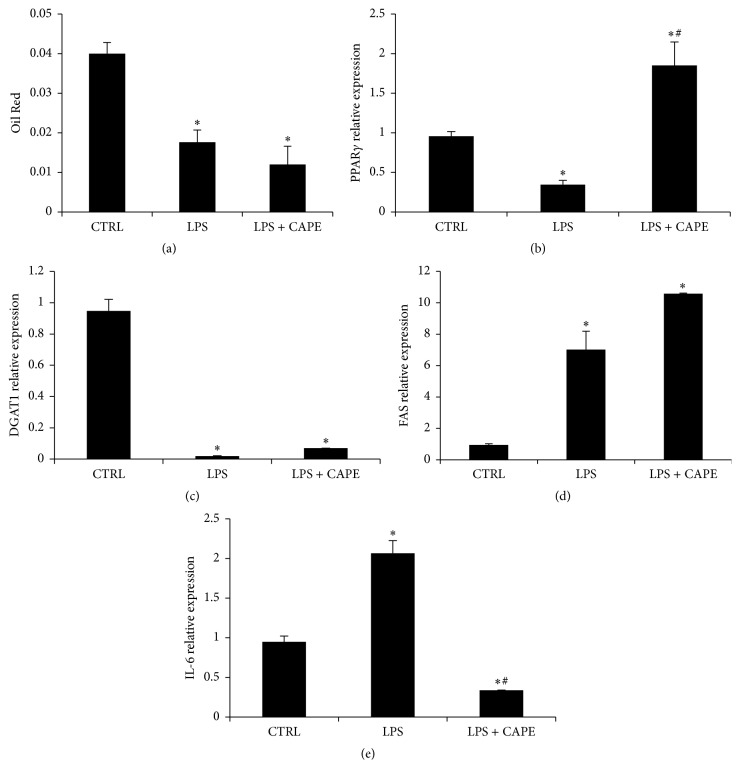
Effect of Cape (10 *μ*M) and LPS on ASC-derived adipocytes. LPS was added 6 hrs before collecting cells at a dose of 1 ng/mL. Values represent the means ± SD of 4 experiments. ^*∗*^ *p* < 0.05, significant result versus differentiated cells (Ctrl). ^#^ *p* < 0.05, significant result versus LPS.

Our results show that Oil Red O staining quantification was decreased after LPS treatment compared to untreated ASCs-derived adipocytes (Figure 7(a)). In agreement with the obtained data, LPS treatment markedly decreased PPAR*γ* and DGAT1 and significantly increased FAS and IL-6 mRNA levels (Figures 7(b)–7(e)). In contrast, in the presence of LPS and Cape, PPAR*γ* and DGAT1 levels were enhanced whereas IL-6 mRNA levels were reduced. Cells exposed to LPS and Cape showed no significant increase of FAS mRNA compared to LPS treatment alone.

These observations suggest that LPS causes induction of insulin resistance, inflammation, and lipolysis of triglycerides stored in adipocytes.

## 4. Discussion

The subcutaneous adipose tissue (SAT) is the largest adipose tissue depot in humans but its ability to expand is limited and, when its storage capacity is exceeded, fat is stored in other metabolically more harmful ectopic lipid depots, such as liver, myocardium, and skeletal muscles [20–22].

Several clinical studies have shown that hypertrophic, rather than hyperplastic, obesity is associated with insulin resistance and dyslipidemia [23, 24].

This study documents a novel prospective on functional adipogenesis that appears to play a central role in providing adipose tissue insulin sensitivity and functionality and in preventing the development of insulin resistance and type II diabetes.

ASCs were isolated from a patient's adipose tissue and, differently from the murine immortalized cell line 3T3, represent a primary culture with a high clinical value for their multipotent properties suitable for tissue engineering and regenerative medical applications.

To investigate the effects of Cape on human adipocyte differentiation, ASCs isolated from the subcutaneous adipose tissue of a human donor were exposed to Cape added to the differentiation medium for 14 days. ASCs accumulated lipid droplets following exposure to the differentiation medium; however, consistent with previous reports on murine cells, adipocyte differentiation, in the presence of Cape, was reduced compared to untreated cells [14]. To assess whether the reduced adipocyte differentiation induced by Cape could be explained by altered activation of typical key markers of mature adipocytes, PPAR*γ*, CEBP*α*, and adiponectin levels were investigated. Figures 2 and 3 show that Cape attenuates both PPAR*γ* and CEBP*α* mRNA and increases adiponectin levels. The latter represents a hormone with insulin-sensitizing, anti-inflammatory, and antiapoptotic functions released by functional adipocytes. It should be noted that the presence of Cape initially decreased and then increased the transcript levels of adiponectin when compared to untreated cells. The initial effect of Cape on adiponectin is associated with a substantial not differentiation of ASCs that do not produce adiponectin. In agreement with Shin et al. [25], the delay in cell cycle progression during cellular differentiation induced by Cape may be due to its antioxidant activity reflected by a reduction of ROS formation and by the increase, after 14 days, of HO-1 mRNA levels. HO-1 catalyzes the rate-limiting step in heme degradation, resulting in the formation of carbon monoxide, iron, and biliverdin [26]. HO-1 represents a key endogenous modulator of oxidative, inflammatory, and cytotoxic stress, exhibiting vasoregulatory properties [27–32]. Upregulation of HO-1 in obesity decreased adiposity and increased adiponectin levels [33, 34]. Given the role of Cape in preventing adipogenesis, finally the present data demonstrate a new function of Cape in the regulation of insulin resistance following an extensive exposure of differentiated adipocytes to high glucose.

To investigate the activation of the genetic program leading to the adipocyte phenotype, mRNA expression levels of gene involved in glucose and lipid metabolism were assessed.

ASCs from the subcutaneous adipose tissue were analyzed at baseline, 7 days, 18 days, and 21 days after induction of adipocyte differentiation.

A good metabolic regulation requires finely balanced control of the capacity of adipose tissue to store and metabolize lipids, responsive to the types and quantity of substrates available.

After 21 days of differentiation, fatty acid synthesis is significantly decreased compared to day 18th as reflected by the reduction of SREBP, FAS, ME1, and SCD mRNA levels. At the same time-point, adipocytes display a reduction of beta-oxidation, evidenced by a twofold of decrease of PPAR*α* (data not shown) and a highly significant increase of triglycerides levels as shown by an increase of DGAT1 mRNA levels.

Adipocyte enlargement due to increased accumulation of triglycerides is associated with an increase in the levels of the proinflammatory cytokine TNF*α*, a decrease of adiponectin, and increased insulin resistance. It has been shown that hypertrophic obesity, through low-grade inflammation, can drive insulin-resistance and type II diabetes [35].

On the other hand, smaller and not hypertrophic adipocytes are considered to be healthy, functional, and insulin-sensitive adipocytes capable of producing adiponectin [36, 37].

The expression of preadipocyte factor-1 (DLK1) was decreased during differentiation, but Cape treatment prevented its downregulation and markedly increased the mRNA levels.

This is an interesting finding because the protein encoded by this gene has been shown to inhibit maturation and lipid synthesis in preadipocytes [38].

As previously reported, PPAR*γ* represents the master gene regulator of adipocyte commitment and its activation leads to improvement of insulin sensitivity [39, 40]. In light of this evidence, we investigated the effect of Cape on PPAR*γ* mRNA levels. The data obtained demonstrate that treatment with Cape restores the function of fully differentiated adipocytes as shown by a significant increase of PPAR*γ*, adiponectin, IRS1, and a concomitant decrease of leptin, TNF*α*, IL-1b, IL-8, IL-6, ICAM-1, and NF*κ*B levels. Proinflammatory environment inhibits adipogenesis and adipocyte insulin response [35].

The improvement in insulin sensitivity, induced by Cape treatment, restored the activity of the transcriptional factor SREBP-1c and the capacity to synthetize fatty acids. It is noteworthy that the elevated levels of leptin observed at day 21 may represent an adaptive response to the decreased levels of adiponectin. Leptin and adiponectin are both anti-inflammatory cytokines released by the adipose tissue but with different target and timing. Only healthy and not inflamed adipocytes produce adiponectin while the pick of leptin is reached by mature hypertrophic adipocyte, as we have shown in our results.

A physiological increase of leptin levels stimulates in the hypothalamus a specific signaling cascade that results in the inhibition of several orexigenic neuropeptides, while stimulating several anorexigenic peptides. Obese patients have high plasma leptin concentrations related to the size of adipose tissue, but the elevated leptin production does not induce the expected responses (i.e., a reduction in food intake and an increase in energy expenditure).

This phenomenon suggests that obese patients are resistant to the effects of endogenous leptin [41]. As shown by Lustig et al. [42], the observed leptin resistance may be attributed to an increase of insulin resistance in obese patients.

When substrates are present in excess, the adipocytes must respond to prevent detrimental accumulation of lipids and the resulting insulin resistance in other tissues. At a molecular level, adipocytes have the ability to synthetize and oxidize fatty acids and to accumulate them as triglycerides. The major physiological role for white adipose tissue fat stores is to supply lipid energy when it is needed by other tissues; this is achieved by a highly regulated pathway whereby the triglycerides stored in the adipocyte are hydrolyzed, and fatty acids are delivered to plasma. Treatment with Cape restores the lost balance between fatty acid synthesis, triglycerides synthesis, lipolysis, and beta-oxidation. A potential player in the relationship between inflammation and insulin resistance is SIRT1 [43–45]. This is a prominent member of the family of NAD^+^-dependent enzymes that deacetylate lysine residues on various proteins such as NF-*κ*B [46]. Interestingly, it has recently been reported that PPAR*δ* activation can increase the expression of SIRT-1 [47]. Inhibition of SIRT-1 downregulated PPAR-*α* gene and decreased fatty acid *β*-oxidation [48]. The results of the present study show that Cape treatment increases beta-oxidation as evidenced by a significant increase of SIRT-1, PPAR*α*, and PPAR*δ* mRNA and decreases lipid accumulation as evidenced by a decrease of DGAT1 levels. The DGAT1 enzyme is expressed mainly in adipocytes and catalyzes the critical final step of triglycerides synthesis [49].

Lipopolysaccharide (LPS) plays pivotal roles in obesity-associated inflammation [50, 51].

The present experiments were therefore performed to assess the LPS-induced effects on lipid metabolism in human ASC-derived mature adipocytes.

LPS has been reported to induce nuclear factor-*κ*B (NF*κ*B) signaling through Toll-like-receptors (TRLs) in macrophages and preadipocytes and has been linked to insulin resistance [52]. However, the mechanism by which LPS induces inflammation and insulin resistance in human ASC-derived adipocytes needs further investigations.

The mRNA levels of PPAR*γ* and DGAT1 were attenuated whereas, as expected, IL-6 levels were increased by LPS treatment. Our results are in agreement with the data published by Chung et al., showing that LPS inhibits PPAR*γ* activity and stimulates lipolysis [53]. Excessive lipolysis contributes to high circulating levels of fatty acids and the development of the dyslipidemia associated with the insulin resistance seen in the metabolic syndrome [54]; a blockade of fatty acid release may be of therapeutic interest. We demonstrated, by inducing acute inflammation with LPS, that Cape treatment restores insulin sensitivity, reduces inflammation, and slightly increases the storage of triglycerides.

Our* in vitro* results support an alternative and additional interpretation recognizing SAT adipogenesis as an active and regulatory mechanism involved in the prevention of accumulation of triglycerides in ectopic sites and the associated insulin resistance.

**Figure 8:**
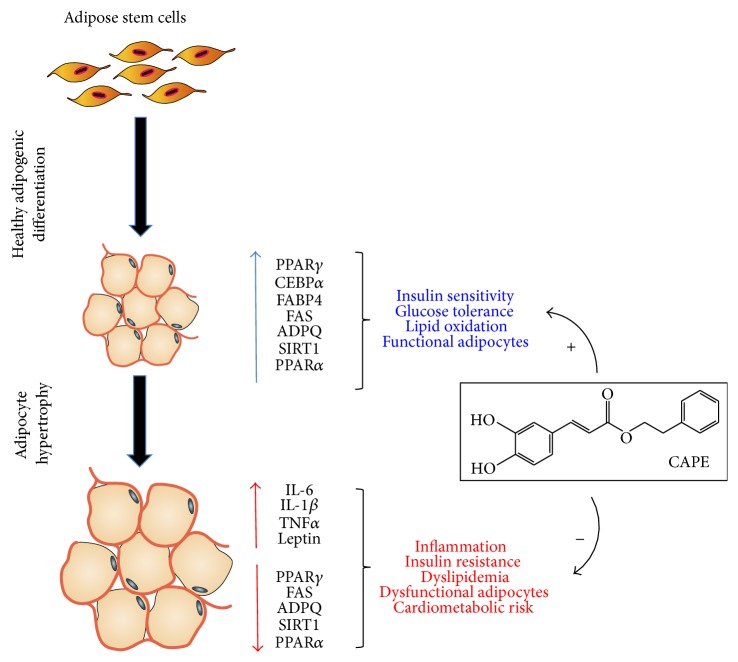
Proposed mechanisms demonstrating the role of Cape in the regulation of ASCs lipid metabolism via induction of PPARs and adiponectin. Cape restores the function of hypertrophic adipocytes by decreasing inflammatory cytokines and increasing insulin sensitive genes.

It is well-established that not all obese patients are insulin resistant and* vice versa* [55]. The mechanism of action of antidiabetic compounds, such as thiazolidinediones (TZD), relies on the ability of these ligands to reduce hepatic lipid content associated with a concomitant increase in subcutaneous fat mass. In the context of TZD, this effect critically depends on their ability to induce PPAR*γ* and adiponectin levels [56, 57]. In conclusion, our data provide direct evidence that Cape treatment restores the function of adipocytes by increasing adiponectin and PPAR*γ* resulting in the reduction of proinflammatory factors (Figure 8).

## Figures and Tables

**Table 1:** PCR primers used in this study.

Gene	Primer forward	Primer reverse
Adiponectin	AGGCTTTCCGGGAATCCAAG	CGCTCTCCTTCCCCATACAC
CEBP*α*	TAACTCCCCCATGGAGTCGG	ATGTCGATGGACGTCTCGTG
DGAT1	CGCGGACTACAAATGGACGA	AACCAGTAAGACCACAGCCG
DLK1	TCCTCAACAAGTGCGAGACC	CTGTGGGAACGCTGCTTAGA
FABP4	AAACTGGTGGTGGAATGCGT	GCGAACTTCAGTCCAGGTCA
FAS	CGGAGGCATCAACCCAGATT	GATGGTGGTGTAGACCTTCCG
GAPDH	AGACACCATGGGGAAGGTGA	TGGAATTTGCCATGGGTGGA
HO-1	GTGCCACCAAGTTCAAGCAG	CACGCATGGCTCAAAAACCA
ICAM1	TCTTCCTCGGCCTTCCCATA	AGGTACCATGGCCCCAAATG
IL1*β*	CCAAACCTCTTCGAGGCACA	AACACGCAGGACAGGTACAG
IL6	CTTCTCCACAAGCGCCTTCG	CTGGCATTTGTGGTTGGGTC
IL8	GGTGCAGTTTTGCCAAGGAG	TTCCTTGGGGTCCAGACAGA
IRS1	GCAACCAGAGTGCCAAAGTG	AGGTCATTTAGGTCTTCATTCTGCT
LEPTIN	TCACACACGCAGTCAGTCTC	AGCTCAGCCAGACCCATCTA
ME1	CGGAACCCTCACCTCAACAA	AGAGACCTCTTGGCTTCCGA
NF*κ*B	TCGGGACTTTCCTAAGCTGC	GAGAGCGAGATCCGGAGTTG
PPAR*α*	AAGAGCTTGGAGCTCGGC	TGAAAGCGTGTCCGTGATGA
PPAR*γ*	AGAGTACGTGGGAGAAATGAC	GATGGCCACCTCTTTGCTCT
PPAR*δ*	GGGACAGGCTGATGGGAAC	TGAACACCGTAGTGGAAGCC
SCD	CTTGCGATATGCTGTGGTGC	CCGGGGGCTAATGTTCTTGT
SIRT1	TGATTGGCACAGATCCTCGAA	AAGTCTACAGCAAGGCGAGC
SREBP-1c	CCCCACTTCATCAAGGCAGA	GCTGTGTTGCAGAAAGCGAA
TNF*α*	CTCGAGTCAGATCATCTTCTCGCACCCCG	GGAATTCTGTTCGTCCTCCTCACAGGGC

## Abbreviations

ASC: Adipose Stem Cells
PPAR*γ*: Peroxisome proliferator-activated receptor gamma
CEBP*α*: CCAAT/enhancer binding protein alpha
FABP4: Fatty acid binding protein 4
SREBP-1c: Sterol regulatory element binding transcription factor 1
ME1: Malic enzyme
SCD: Stearoyl-Coa desaturase-1
FAS: Fatty acid synthase
SIRT1: Sirtuin 1
IRS1: Insulin receptor substrate 1
ICAM1: Intercellular adhesion molecule 1
TNF*α*: Tumor necrosis factor alpha.
